# Emotion and Stress Recognition Related Sensors and Machine Learning Technologies

**DOI:** 10.3390/s21072273

**Published:** 2021-03-24

**Authors:** Kyandoghere Kyamakya, Fadi Al-Machot, Ahmad Haj Mosa, Hamid Bouchachia, Jean Chamberlain Chedjou, Antoine Bagula

**Affiliations:** 1 Institute for Smart Systems Technologies, Universitaet Klagenfurt, A9020 Klagenfurt, Austria; ahmad.haj.mosa@pwc.com (A.H.M.); Jean.Chedjou@aau.at (J.C.C.); 2 Department of Applied Informatics, Universitaet Klagenfurt, 9020 Klagenfurt, Austria; Fadi.AlMachot@aau.at; 3 Machine Intelligence Group, Bournemouth University, Bournemouth BH12 5BB, UK; abouchachia@bournemouth.ac.uk; 4 ISAT Laboratory, University of the Western Cape, 7535 Bellville, South Africa; bbagula@uwc.ac.za

Intelligent sociotechnical systems are gaining momentum in today’s information-rich society, where different technologies are used to collect data from such systems and mine this data to make useful insights about our daily activities. These systems range from driver-assistance systems, to medical-patient monitoring systems, to emotion-aware intelligent systems, to complex collaborative robotics systems. They are built around (i) intrusive technologies such as physiological sensors, used for example in EEG, ECG, electrodermal activity and skin conductance and (ii) nonintrusive technologies that use piezo-vibration sensors, facial images, chairborne differential vibration sensors and bed-borne differential vibration sensors. However, despite their undisputable advantages in our daily lives, there are a number of issues relating to the design and development of such systems, as they rely on emotion and stress classification from physiological signals. These issues can be viewed from various perspectives including: (a) quality and reliability of sensor data; (b) classification performance in terms of accuracy, precision, specificity, recall and F1-measure; (c) robustness of subject-independent recognition; (d) portability of the classification systems to different environments and (e) the estimation of the emotional state for dynamic systems.

This book emerging from the Special Issue of the *Sensors* journal on Emotion and Stress Recognition Related Sensors and Machine Learning Technologies emerges as a result of the crucial need for massive deployment of intelligent sociotechnical systems. Such technologies are being applied in assistive systems in different domains and parts of the world to address challenges that could not be addressed without the advances made in these technologies. The Special Issue includes 25 papers submitted in response to the call for papers. The high number of submissions to the Special Issue is an indication of the momentum of the current research in this field. This momentum is driven not only by technological development, but also the need for assistive technologies. The Special Issue includes impactful papers that present scientific concepts, frameworks, architectures and ideas on sensing technologies and machine-learning techniques. These are relevant in tackling the following challenges: (i) the field readiness and use of intrusive sensors systems and devices for capturing biosignals, including EEG sensor systems, ECG sensor systems and Electrodermal activity sensor systems; (ii) the quality assessment and management of sensor data; (iii) data preprocessing, noise filtering and calibration concepts for biosignals; (iv) the field readiness and use of nonintrusive sensor technologies, including Visual sensors, Acoustic sensors, Vibration sensors and Piezo-electric sensors; (v) emotion recognition using mobile phones and smartwatches; (vi) body area sensor networks for emotion and stress studies; (vii) the use of experimental datasets in emotion recognition, including datasets generation principles and concepts, quality insurance and emotion elicitation material and concepts; (viii) machine-learning techniques for robust emotion recognition, including Graphical models, Neural network methods, Deep learning methods, Statistical learning and Multivariate empirical mode decomposition; (ix) subject-independent emotion and stress recognition concepts and systems, including Facial expression-based systems, Speech-based systems, EEG-based systems, ECG-based systems, Electrodermal activity-based systems, Multimodal recognition systems and Sensor fusion concepts and (x) emotion and stress estimation-and-forecasting from a nonlinear dynamical system’s perspective.

In general, these papers are grouped into four categories/groups:

1. Stress level recognition
2. Wearable body sensors
3. Dermatological sensors
4. Facial expression recognition

## 1. Stress Detection

Addressing the issue of stress as a naturally occurring psychological response, identifiable by several body signs, [1] proposed a novel way of discriminating between acute stress and relaxation by using movement and posture characteristics of the foot. The authors used several machine-learning techniques to build models that were used to assess the validity of their method based on data collected from 23 participants performing tasks that induced stress and relaxation. Data collected from an additional sample of 11 participants were used to test their models, with results demonstrating replicability and an overall accuracy of 87%. External validity was also demonstrated by conducting a field study with 10 participants that revealed the robustness of the results.

The research in [2] contributed to bridging the gap between laboratory experimentation and daily life activities. The authors used a laboratory experiment and ecological momentary assessment-based data collection with smartwatches in daily life to propose a stress level detection system. The system pre-processes noisy physiological signals, extracts features and applies machine-learning techniques to classify the levels of stress. The study revealed that the accuracy of the system when tested in daily life improved significantly when machine-learning models were trained in the laboratory instead of with data from daily life.

In [3], regression and classification models were compared for stress detection using both personal and user-independent models’ experimentation. The paper used the stress-detection dataset AffectiveROAD, which contained data gathered using Empatica E4 sensor and also continuous target variables—a feature that is missing in the other stress-detection dataset. The two classification models used for stress detection were Random Forest and Bagged tree based ensemble. From conducted experiments and using the AffectiveROAD dataset, the study revealed that regression models outperform classification models when classifying observations as stressed or not-stressed.

The research done in [4] revisited stress by using EEG as an objective measure for cost-effective and personalized stress management in situations where mental health facilities are not available. The study conducted by the paper considered: (i) a scenario in which- long-term stress was classified with machine-learning algorithms using resting state EEG signal recordings and (ii) the labelling for the stress and control groups was performed using two currently accepted clinical practices: the perceived stress scale score and expert evaluation. Support vector machine was found by the authors to be the most suitable classification algorithm for long-term human stress when used with the alpha asymmetry feature.

## 2. Wearable Body Sensors

The main contribution of [5] was to study electroencephalography (EEG) and galvanic skin response (GSR) together for boredom classification, with the objective of using the potential features of the associated data for emotion classification. The authors investigated the combined effect of these features on boredom classification by: (i) collecting EEG and GSR data from 28 participants using off-the-shelf sensors; (ii) labelling the collected samples using the participants’ questionnaire-based testimonies of the various boredom levels experienced; (iii) using the collected data to initially train 30 models with 19 machine-learning algorithms and select the top three candidate classifiers and (iv) tuning the hyperparameters and validating the final models through 1000 iterations of 10-fold cross validation to increase the robustness of the test results. The work revealed the relative efficiency of multilayer perceptron compared to other machine-learning techniques. It also showed the correlation between boredom and the combined features of EEG and GSR.

The research in [6] addressed the issues of features extraction from Electroencephalography (EEG) signals and emotional aspects by considering both intra-subject and inter-subject approaches to EEG-based affect detection. Using three public repositories, the paper analysed both modelling approaches and showed that the subject’s influence on the EEG signals is substantially higher than that of the emotion, thus (i) the subject’s influence on the EEG signals should be accounted for and (ii) a data transformation that seamlessly integrates individual traits into an inter-subject approach should be performed to improve the classification process.

In [7], the authors suggested a better classification method for detecting stressed states based on raw electrocardiogram (ECG) data and a method for training a deep neural network (DNN) with a smaller data set. The work built an end-to-end architecture to detect stress using raw ECGs, using a multistage architecture that includes convolutional layers. Two kinds of datasets were used to train and validate the model, which were: a driving dataset and a smaller mental arithmetic dataset. A transfer learning method was then used to train the proposed model with a small dataset. It is shown in the paper that: (i) based on receiver operating curves, the proposed model performs better than conventional methods and (ii) compared with other DNN methods using raw ECGs, both the proposed model and the transfer learning method improves accuracy. These findings revealed that the proposed model can significantly contribute to mobile healthcare for stress management in daily life.

The issue of recognizing mental stress with deep ECG-respiration network was addressed in the workplace by proposing a novel stress-detection algorithm that uses multiple physiological signals, such as electrocardiogram (ECG) and respiration (RESP) signals to achieve end-to-end deep learning in [8]. The study mimicked workplace stress by using Stroop and mathematical tasks as stressors, with each stressor being followed by relaxation task(s). It also provided experimental results demonstrating its superiority over conventional machine-learning models.

The authors in [9] focused on the field readiness of low-cost wearable devices, which are increasingly being used in research as well as for personal and private purposes. The goal was to evaluate the accuracy of these devices in comparison to well-calibrated, high-quality devices used in laboratory experiments for physiological and medical research. The study demonstrated an approach for quantification of the accuracy of low-cost wearables in comparison to high-quality laboratory sensors by developing a benchmark framework for physiological sensors. The benchmark covered the entire workflow from sensor data acquisition to computation and interpretation of diverse correlation and similarity metrics. The study showed that the benchmarked wearables provide physiological measurements, such as heart rate and interbeat interval, with an accuracy close to those of the professional/high-end sensors. It was also revealed that accuracy varied more for parameters such as galvanic skin responses.

In [10], the issue of remote patient monitoring was revisited with the perspective of developing a wearable device that was low cost, single channel, dry contact and suitable for in-ear EEG for nonintrusive monitoring. The paper covered all aspects of the designs, engineering and experimenting. By applying machine learning for emotion classification, it was revealed that the proposed device was able to classify basic emotion with results that were comparable to those measured from the more conventional EEG headsets at T7 and T8 scalp positions.

In [11], a deep analysis of features proposed to extract information from the electrocardiogram, thoracic electrical bioimpedance and electrodermal activity signals was carried out with a focus on activities such as neutral, emotional, mental and physical. The study tested a total of 533 features for activity recognition. A comprehensive study was then performed taking into consideration the prediction accuracy, feature calculation, window length and type of classifier. This study enabled the determination of the ideal number of features and the best subset of features among those proposed in literature to obtain good error probability while avoiding over-fitting.

## 3. Dermatological Sensors

The association between the physiological responses of a driver and driving stress was addressed in [12], where the relationship between driving stress and traffic conditions, and driving stress and road types, respectively, was quantified through research. The study used electrodermal activity (EDA) signals for a male driver collected in real road-driving conditions for 60 min a day and over a 21-day period. Two separate models were used that incorporate the statistical features of the EDA signals, one for traffic conditions and the other for road types to classify the levels of driving stress (low vs. high). The classification results of the two models indicated that the traffic conditions and the road types were important features for driving stress and its related applications.

The work done in [13] addressed the issue of Active and Assisted Living environments for elderly and/or disabled people and the subjectivity of results when training a machine-learning model on a specific group of people while testing on a totally new group of persons. The study relied on electrodermal activity sensors to collect emotions and used a Convolutional Neural Network (CNN) architecture to provide promising robustness-related results for both subject-dependent and subject-independent human emotion recognition. The results revealed that by solely using the nonintrusive EDA sensors, a robust classification of human emotion was possible even without involving additional/other physiological signals.

The research in [14] presented the identification of the level of arousal in older people by monitoring their electrodermal activity (EDA) through a commercial device. The objective was to use the notion of familiarity with a musical genre on emotional induction in order to recognize arousal changes and hence create future therapies that can help older people to improve their mood. This can ultimately contribute to the reduction of depression and anxiety. Using methods based on the process of deconvolution of the EDA signal, two different studies were carried out, the first being a purely statistical study based on the search for statistically significant differences for a series of temporal, morphological, statistical and frequency features of the processed signals. The second study was a machine-learning study using a wide range of classifiers to analyse the possible correlations between the detection of the EDA-based arousal level compared to the participants’ responses to the level of arousal subjectively felt. While the first study revealed that Flamenco and Spanish Folklore presented the highest number of statistically significant parameters, the second study showed that the best classifiers are the support vector machines, with 87% accuracy for Flamenco and 83.1% for Spanish Folklore, followed by K-nearest neighbours.

Motivated by the limitations of emotion recognition systems in terms of lack of systematic analysis in literature regarding the selection of classifiers to use, sensor modalities, features and range of expected accuracy, and many other limitations, the work in [15] contributed to the body of work in machine learning by presenting a systematic study across five public datasets commonly used in Emotion Recognition (ER) with the objective of evaluating emotion in terms of low/high arousal and valence classification through Supervised Learning (SL), Decision Fusion (DF) and Feature Fusion (FF) techniques using multimodal physiological data, namely Electrocardiography (ECG), Electrodermal Activity (EDA), Respiration (RESP) or Blood Volume Pulse (BVP). The work considered: (i) Classification performance analysis of ER benchmarking datasets in the arousal/valence space; (ii) Summarising the ranges of the classification accuracy reported across the existing literature; (iii) Characterising the results for diverse classifiers, sensor modalities and feature set combinations for ER using accuracy and F1-score; (iv) Exploration of an extended feature set for each modality and (v) Systematic analysis of multimodal classification in DF and FF approaches. The study revealed that FF is the most competitive technique in terms of classification accuracy and computational complexity.

Moving away from the affective computing research that has mostly used nonimmersive two-dimensional (2D) images or videos to elicit emotional states, [16] adopted an immersive virtual reality (VR) approach. This allowed the researchers to simulate various environments in controlled laboratory conditions with high levels of sense of presence and interactivity. The paper presented a systematic review of the emotion recognition research undertaken with physiological and behavioural measures using head-mounted displays as elicitation devices. The results highlighted the evolution of the field, gave a clear perspective of the use of aggregated analysis and revealed the current open issues and guidelines for future research works.

Focusing on affecting computing, which is an artificial intelligence area of study that recognizes, interprets, processes and simulates human affect computers, a survey of the pertinent scientific literature on affecting computing from 2015 to 2020 was presented in [17]. The paper presented trends and compared algorithm applications in new implementations from a computer science perspective. The survey provided an overview of datasets, emotion elicitation methods, feature extraction and selection, classification algorithms and performance evaluations.

## 4. Facial Expression Recognition

Building upon deep transfer learning techniques, facial expression recognition (FER) was addressed in [18]. The authors tackled the challenging issues of: (i) diversity of factors, which are unrelated to facial expressions (ii) the lack of training data for FER and (iii) the intrinsic imbalance in existing facial emotion datasets. The deep transfer contribution to FER was complemented by a novel loss function called weighted-cluster loss used during a fine-tuning phase of the model.

In [19], the authors revisited the analysis of pain-related facial expressions by proposing an end-to-end approach based on attention networks for the analysis and recognition of pain-related facial expressions. The method proposed by the authors combined both spatial and temporal aspects of facial expressions through a weighted aggregation of attention-based neural networks’ outputs that use sequences of Motion History Images (MHIs) and Optical Flow Images (OFIs). A combination of Convolutional Neural Network (CNN) and Bidirectional Long Short-Term Memory (BiLSTM) Recurrent Neural Network (RNN) was used to achieve pain recognition.

Building around a human-computer interaction (HCI) setting, [20] addressed the challenging issue of induction of dialog-based HCI relevant emotional and cognitive load states by presenting a multimodal dataset for affective computing research. The dataset used an experimental mobile and interactive scenario design that was implemented based on a gamified generic paradigm. The work consisted of six experimental sequences inducing Interest, Overload, Normal, Easy, Underload and Frustration.

Facial-landmark detection was revisited in [21] in a multistage architecture. At the first stage, the goal was to obtain local pixel-level accuracy for local-context information. The second stage was concerned with integrating obtained information with knowledge of spatial relationships between each key point in a whole image for global-context information. The paper considered a pipeline architecture consisting of two main components: (i) a deep network for local-context subnet used to generate detection heatmaps via fully convolutional DenseNets with additional kernel convolution filters and (ii) a dilated skip convolution subnet consisting of a combination of dilated convolutions and skip-connections networks used to robustly refine the local appearance heatmaps.

Building around the Child–Robot Interaction (CRI), [22] proposed a system for emotion recognition in children by recording facial images using both visual (RGB—red, green and blue) and Infrared Thermal Imaging (IRTI) cameras. Building upon the Viola–Jones algorithm on colour images to detect facial regions of interest (ROIs), the paper proposed as a novel contribution the computation of the error probability for each ROI located over thermal images, using a reference frame manually marked by a trained expert, in order to choose that ROI better-placed according to the expert criteria. The results: (i) show that the proposed approach for ROI locations may track facial landmarks with significant low errors with respect to the traditional Viola–Jones algorithm and (ii) suggest that the proposed system be integrated to a social robot to infer child emotions during a child–robot interaction.

A comparison of machine-learning algorithms applied to the recognition of emotion intensities was proposed in [23] as a solution to the lack of encoding the intensity of observed facial emotion and multifacial behaviour in existing emotion recognition systems. The work compared several algorithms, include (i) Gabor filters, a Histogram of Oriented Gradients (HOG), and Local Binary Pattern (LBP) for feature extraction and (ii) Support Vector Machine (SVM), Random Forest (RF), and Nearest Neighbour Algorithm (KNN) for classification. The experiment suggested that the comparative study could be further used in real-time behavioural facial emotion and intensity of emotion recognition.

A transfer learning approach was adopted for mouth-based emotion recognition in [24]. The study was predicated on the fact that there were only a few datasets available in practice and most of them included emotional expressions simulated by actors, instead of adopting real-world categorisation. By enabling the image of the mouth to be available, even when the whole face was only visible from an unfavourable perspective, the transfer learning approach allowed the authors to use fewer training data. This minimized the effort of training a whole network from scratch and resulted in an improved dynamic emotion recognition when taking into account not only new scenarios but also modified situations to the initial training phase. As presented in the paper, the transfer learning approach and the underlying method proved the relevance of mouth detection in the complex process of emotion recognition.

The authors in [25] proposed a multimodal approach to emotion recognition in the aviation domain with the goal of filling some of the gap between pilots’ emotions and their bioreactions during flight procedures such as take-off, climbing, cruising, descent, initial approach, final approach and landing. Building around a sensing architecture and a set of simulated flight experiments, the study showed that it was indeed possible to recognize emotions from different pilots in flight, combining their present and previous emotions.

As we alluded to in our introduction, assistive technology is a research field with a number of open challenges. Some of those are present in this Special Issue, which we think will foster more research. Other fields were not covered, hence leaving room for new ideas to be discovered in this field.
